# Can editors save peer review from peer reviewers?

**DOI:** 10.1371/journal.pone.0186111

**Published:** 2017-10-09

**Authors:** Rafael D’Andrea, James P. O’Dwyer

**Affiliations:** Dept of Plant Biology, University of Illinois, Urbana-Champaign, Illinois, United States of America; Universita degli Studi di Ferrara, ITALY

## Abstract

Peer review is the gold standard for scientific communication, but its ability to guarantee the quality of published research remains difficult to verify. Recent modeling studies suggest that peer review is sensitive to reviewer misbehavior, and it has been claimed that referees who sabotage work they perceive as competition may severely undermine the quality of publications. Here we examine which aspects of suboptimal reviewing practices most strongly impact quality, and test different mitigating strategies that editors may employ to counter them. We find that the biggest hazard to the quality of published literature is not selfish rejection of high-quality manuscripts but indifferent acceptance of low-quality ones. Bypassing or blacklisting bad reviewers and consulting additional reviewers to settle disagreements can reduce but not eliminate the impact. The other editorial strategies we tested do not significantly improve quality, but pairing manuscripts to reviewers unlikely to selfishly reject them and allowing revision of rejected manuscripts minimize rejection of above-average manuscripts. In its current form, peer review offers few incentives for impartial reviewing efforts. Editors can help, but structural changes are more likely to have a stronger impact.

## Introduction

Peer review is the main process by which scientists communicate their work, and is widely regarded as a gatekeeper of the quality of published research [1]. However, its effectiveness remains largely assumed rather than demonstrated [2]. Despite recent calls for transparency and initiatives to share peer review information [3], there are still no large-scale data to empirically evaluate the process in detail. As a result, the fairness, reliability, transparency, and sustainability of peer review have been repeatedly questioned [4–9], with emphasis on a slew of potential biases by all parties involved [10]. Equally long as the litany of debated issues is the list of proposed solutions, including alternatives to traditional peer review such as preprint repositories [11], double-blind peer review [12], and open peer review [13–15]. Many of those ideas have in turn not been definitively shown to outperform peer review [16], and are met with skepticism from proponents of classical peer review [17, 18]. (For overviews of the history and the current debate on peer review, see [10, 18–20]).

Given the scarcity of empirical data, modeling approaches are increasingly used to test peer review (e.g. [21–25]), with particular attention devoted to referee behavior. In particular, the reliance of peer review on the availability, good will, and impartiality of reviewers has been recently shown to be a potentially severe hurdle to quality control [26–29]. If these results prove robust in the face of uncertainties from lack of data, they can be instrumental for improving the system.

Reviewers are typically protected by anonymity, and are not rewarded for an accurate and fair job nor held accountable for a sloppy or biased one. Reviewers are thus under little incentive to act in the best interest of science as opposed to their own best interest. Thurner and Hanel [26] showed that the average quality of published research suffers substantially if even a small percentage of referees adopt “rational” (i.e. selfish) behavior, rejecting manuscripts they perceive to threaten the visibility of their own work. Relative to a scenario where all referees are fully impartial, a percentage as low as 10% of selfish referees in the pool will lower the quality of published work by almost one full standard deviation of the quality of submitted manuscripts.

Although striking, Thurner and Hanel’s [26] results are due in part to the extreme behavior adopted by both impartial and selfish referees in their model: impartial referees are constantly raising the bar on manuscripts they will accept, while selfish referees accept manuscripts of below-average quality. A scenario with only impartial referees quickly results in no papers getting published anymore because the standards are so high; in contrast, a scenario with only selfish referees results in the average published paper being of lower quality than the average submitted manuscript. Both outcomes seem too radical.

A second limitation of Thurner and Hanel’s [26] model is that it attributes no moderating power to editors, who in reality are expected to buffer selfish referee behavior in several ways. For example, editors may match manuscripts to suitable referees, aiming to avoid bias caused by conflicts of interest and cliques [22]. Editors may also send manuscripts to multiple referees; [24] showed that this also reduces bias, albeit at the expense of more resources invested by scientists on reviewing rather than researching. Editors may bypass reviewers altogether when manuscripts are exceptionally good or bad, accepting or rejecting without review as the case may be [30]. Editors also typically give authors the chance to revise their manuscripts to address reviewers’ comments, and may even blacklist referees with a suspicious reviewing record.

Finally, peer review must balance its goal of guaranteeing quality of published work with other desired outcomes, such as minimizing turnaround times, meeting increasing demand from continued growth of scientific production [8, 9], keeping rejection of good papers at a minimum, and distributing the reviewing load evenly across scientists ([22, 31]. As few as 20% of researchers may be responsible for over 90% of the reviews, see [9]).

Here we extend Thurner and Hanel’s [26] agent-based model of peer review to examine how their results change when referees adopt less extreme behavior, such as when impartial referees adopt fixed quality standards and selfish referees care enough about quality control to reject below-average papers. We then ask to what degree the aforementioned editorial strategies can mitigate the negative impact of selfish referees on the average quality of published work. We note that other authors have tested some of these editorial strategies [24, 28], but ours is the first study to examine them in concert with different types of referee behavior. We also investigate how editors and referees affect the proportion of above-average manuscripts that go unpublished, and the distribution of reviewing effort among all scientists.

## Methods

### The peer review process

Thurner and Hanel [26] (T&H) proposed an agent-based model of peer review consisting of cycles where authors submit manuscripts to journals and each manuscript is either rejected or accepted for publication. Each manuscript has a single author, and is handled by an editor and two randomly selected referees. Each referee either accepts or reject the manuscript, and the editor makes the final call based on the referee assessments. If the referees are unanimous, the editor honors their verdict. If they disagree, the editor accepts it with 50% probability. T&H assume a fixed pool of scientists variously acting as authors and referees, and perform a fixed number of iterations of the review cycle before stopping the process.

Manuscripts differ by “quality”, abstractly defined as the property that the peer review process aims to maximize in accepted papers. Quality thus includes elements such as clarity, technical correctness, importance and relevance, interest to readers, etc. Authors differ by the average quality of their manuscripts. T&H define two types of referee, which we refer to as impartial and selfish. T&H’s impartial referees accept a manuscript if and only if it exceeds a minimum quality threshold (detailed below). Selfish referees also have minimum quality standards (which are lower than those of impartial referees), but will reject any manuscript with quality above their own average paper. This behavior represents scientists sabotaging work that they perceive to threaten their own.

Our model extends T&H by introducing different types of impartial and selfish referees, turnover in the scientist pool as old scientists retire and are replaced by new hires, and editorial strategies to minimize the negative impact of selfish referees.

Following T&H we assume a fixed-size scientific community with *N* = 1,000 scientists. Every year there are two peer review cycles, for a total of 500 review cycles in the simulation. In each cycle, half of the scientists submit manuscripts for review (each author submits a manuscript every other cycle). We introduce a turnover of scholars: each scientist remains active for 35 years (70 review cycles) before retiring, and upon retirement is immediately replaced by a new scientist with proficiency randomly drawn from the distribution described below. The new scientist is permanently assigned either selfish or impartial referee behavior, with probabilities *p* and 1 − *p* respectively. Parameter *p* therefore determines the average percentage of selfish referees in the pool.

Let the “proficiency” of an author be the average quality of their papers. We assume author proficiency is a normally distributed random number with mean *μ*_a_ and standard deviation *σ*_a_. Further, the quality of manuscripts by an author with proficiency *Q* is a normally distributed random variable with mean *Q* and standard deviation *σ*_m_. Following T&H, we set *μ*_a_ = 100, *σ*_a_ = 10, *σ*_m_ = 5.

### Referee behavior

Impartial referees base their review strictly on the quality of the manuscript. Selfish referees also consider how the manuscript compares with their own research. Like impartial referees, selfish referees impose a minimum quality threshold. Unlike impartial referees, they also impose an upper limit, namely the average quality of their own scientific output. A selfish referee whose papers have mean quality *Q* will therefore reject any paper with quality greater than *Q*.

We consider two types of impartial referees and two types of selfish referees:

*Fixed-standard* impartial referees accept manuscripts meeting a fixed minimum standard *Q*_min_, and reject all other manuscripts. For concreteness we define this threshold as the 90% quantile of quality distribution across submitted manuscripts, which in our model is a fixed quantity, $Q_{\text{min}}=\mu_{a}+1.28\;\sqrt{\sigma_{a}^{2}+\sigma_{m}^{2}}=114.3$.

*Moving-standard* impartial referees are similar, except their minimum cutoff is based on the average quality of accepted papers in the previous review cycle, and as such is continually updated. Following [26], we define it as $Q_{\text{min}}\left(t\right)=\lambda \;Q_{\text{min}}\left(t-1\right)+\left(1-\lambda \right)\;{\bar{Q}}_{\text{accepted}}\left(t-1\right)$, where *t* is the index of the current review cycle, and *λ* is a fixed parameter controlling how quickly the moving standard asymptotes. We set *λ* = 0.1.

*Conscientious* selfish referees share their minimum standards with fixed-standard impartial referees, *Q*_min_ = 114.3. The only difference is that they are selfish, as defined above.

*Indifferent* selfish referees are less particular about the quality of the manuscripts they review, and will accept below-average manuscripts down to $\epsilon \;{\bar{Q}}_{\text{submitted}}$, where *ϵ* < 1. For consistency with Thurner and Hanel we set *ϵ* = 0.9, leading to a minimum cutoff *Q*_min_ = 90.

For a summary of the minimum and maximum cutoffs of our different types of referees, see Table 1.

**Table 1:** Minimum and maximum cutoffs imposed by each type of referee. Impartial referees have no maximum cutoff, and accept any manuscript above their minimum standard. The two types differ by whether that minimum is fixed or moving as the review process proceeds. A selfish referee whose average work quality is *Q* will reject any manuscript above that mark. Indifferent selfish referees care little about quality of published research, and accept manuscripts of below-average quality. In contrast, conscientious selfish referees share minimum standards with fixed-standard impartial referees. ${\bar{Q}}_{\text{accepted}}$ asymptotes to ≈ 141 when the referee pool consists almost entirely of moving-standard impartial referees, and to gradually lower values as more selfish referees are added.

Referee type	Minimum cutoff	Maximum cutoff
Impartial, moving-standard	${\bar{Q}}_{\text{accepted}}\left(t-1\right)$	∞
Impartial, fixed-standard	114.3	∞
Selfish, conscientious	114.3	*Q*
Selfish, indifferent	90	*Q*

In addition to the referee types above, we also consider the narcissist. That referee has a (conscious or unconscious) bias towards endorsing the relevance or importance of manuscripts on their subfield of expertise. Ironically, this leaves it entirely to the editor to judge the true importance/relevance of a manuscript, a task they may not be best qualified for compared with the more specialized referee. Assuming different topics can be ranked by importance, we model the narcissistic referee as accepting all papers within the scope of importance of their own work, while rejecting all others. We assume importance has the same properties as quality, i.e. it is an objective quantity varying across manuscripts, which referees can accurately assess and peer review aims to maximize.

We note that in this simplified model, referees and editors can accurately assess the quality of a manuscript. As a result, two referees with the same quality standards will always agree on their opinion of a paper; in particular, two impartial referees never disagree. In reality, of course, quality is a complex and subjective concept which will not be readily agreed upon by everyone, and honest referees may earnestly disagree in their assessments. However, the personal agendas of selfish referees will still cause more disagreements than expected between purely impartial referees.

### Editor strategies

In T&H’s model, the editor accepts a manuscript if both referees accept it, rejects it if both referees reject it, and accepts it with probability 50% if the referees disagree. In our model, the editor tries to guarantee the quality of published papers by adopting one the following strategies:

*Consulting a tiebreaking referee*. If one referee accepts while the other rejects, the editor sends the manuscript for review by a third referee, whose recommendation breaks the tie. An equivalent interpretation is that the editor consults three referees from the beginning and decides in favor of the majority.

*Bypassing referees*. Editors automatically reject a manuscript without review if it fails to meet a minimum standard of quality (here we use the 50th percentile of quality among submitted manuscripts). This is commonly done by high-profile journals such as Science and Nature. Similarly, editors accept without review manuscripts deemed to be of exceptional quality (above the 90th percentile of submissions).

*Blacklisting selfish referees*. If a referee’s record of reviews indicates that the referee is selfish, the editor removes that person from the list of referees and no longer sends papers for them to review. Because two impartial referees will never disagree in their reviews but an impartial and a selfish referee or two selfish referees might, a high proportion of disagreements in someone’s reviewing record suggests selfishness. A referee is blacklisted when the probability of selfishness given their reviewing record exceeds a threshold *p*_0_. Our model is simple enough that this conditional probability can be worked out exactly, see S1 Appendix. To a good approximation, the referee is likely selfish if he or she disagrees with the other referee at least half the time.

*Matching manuscript quality to referees*. Aware that some referees tend to selfishly reject better papers than their own, editors preferentially match manuscripts to referees with somewhat better scientific output.

*Returning papers to authors for revision*. If at least one referee rejects the manuscript, the editor sends it back to the author for revision and gives it another round of reviewing by the same referees. In our model, the quality increment of the revised manuscript follows a half-normal distribution with mean $\sqrt{2/\pi }\;\sigma_{m}≃4$.

We assume all journals have the same quality standards and all editors adopt the same editorial strategy in each of our scenarios. In the blacklisting scenario, reviewing records are shared among all editors. These best case scenarios provide insight into these strategies’ maximum potential.

## Results

### Impact of referee behavior

**Fig 1:**
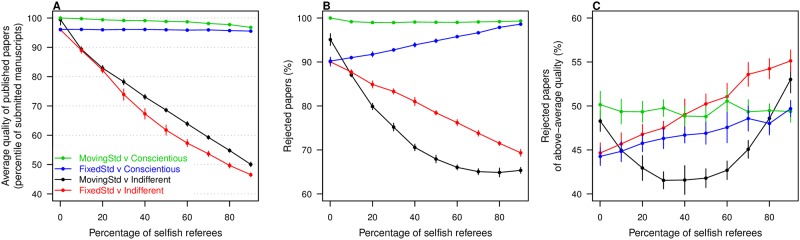
Effect of referee behavior. **A**: Average quality of published papers, in percentile of the quality distribution across submitted manuscripts, when impartial and selfish referees are modeled in different ways. impartial referees have either *moving standards* or *fixed standards*, and selfish referees are either *conscientious* or *indifferent* (see main text for detailed description). Curves show the decline of published quality as the percentage of selfish referees in the pool increases, for each of the four combinations with one type of impartial referee and one type of selfish referee. Thurner and Hanel’s [26] results correspond to the black curve. **B**: Percentage of rejected manuscripts by percentage of selfish referees in the pool, for each of the four scenarios. **C**: Percentage of rejected manuscripts of above-average quality. Error bars represent one standard deviation across ten replicates. Color scheme is consistent across panels.

When all scientists act as moving-standards impartial referees, the average quality of published papers approaches the very top percentile of submitted manuscripts (Fig 1A). Only the very best manuscripts are accepted, with a rejection rate close to 100% (Fig 1B), with around 50% of rejected papers being of higher quality than the average submitted manuscript (Fig 1C). By comparison, fixed-standards impartial referees keep published research at the top 95 percentile of submitted work and reject 90% of manuscripts, about 45% of which are of above-average quality.

The introduction of indifferent selfish referees, which will accept papers of below-average quality (down to *Q* = 90 given our parameter choices), has a strong impact on the quality of published papers and on rejection rates. As the percentage of indifferent selfish referees increases from near 0 to 90%, mean quality of published work falls from near the 100th percentile of submitted manuscripts to the 50th percentile—thus indistinguishable from a fully random review process. The drop is similar whether the impartial referees have moving or fixed standards (compare black and red curves on Fig 1A).

In stark contrast, the quality of published papers does not suffer nearly as much when selfish referees are conscientious, that is when they share the impartial referees minimum quality standards. As more referees in the pool are selfish but conscientious, mean published quality drops very little or none at all, depending on whether impartial referees have moving or fixed standards (green and blue curves on Fig 1A).

Overall rejection rates are relatively low when indifferent selfish referees are common, and high otherwise (Fig 1B). Given our parameter choices, impartial referees of both kinds and conscientious selfish referees will all tend to reject at least 90% of manuscripts. Indifferent selfish referees have the greatest impact on rejection rates because of their tolerance for low-quality papers.

As selfish referees become more common, so does rejection of good papers (Fig 1C). Comparing Fig 1B and 1C, we notice that as indifferent selfish referees become more common, overall manuscript rejection declines but the percentage of rejected papers with above-average quality increases. When selfish referees are conscientious, above-average papers always constitute less than 50% of rejections. But when they are indifferent and dominate the referee pool, more than one in two rejected papers may be in the upper half of submissions.

These results show that the big impact T&H reported for selfish referees is due not to their selfishness but to their indifference. That is, they lower the average quality of accepted literature not because they reject papers whose quality is too high, but because they accept papers whose quality is too low. Selfishness is a bigger problem if the goal is to prioritize publication of excellent papers over raising average published quality. Notice that whether impartial reviewers have fixed or moving standards is relatively inconsequential.

As for narcissistic referees, they have a very similar impact on the importance of published papers as indifferent selfish referees on published quality (S1 Fig). However, they impose a lower cost in terms of rejected papers, and in particular good papers.

Our findings are relatively insensitive to the choice of two referees per paper. Outcomes are qualitatively similar when three rather than two referees are assigned per manuscript, with three referees being overall slightly better than two (S2 Fig). Results are also similar when the quality and proficiency distributions are lognormal rather than normal (see S3 Fig).

Because of the high impact caused by indifference compared to other referee behaviors, we are going to focus on it for the remainder of our study. In the following section, selfish referees are assumed indifferent and impartial referees are assumed to have moving standards, similar to T&H.

### Impact of editors

**Fig 2:**
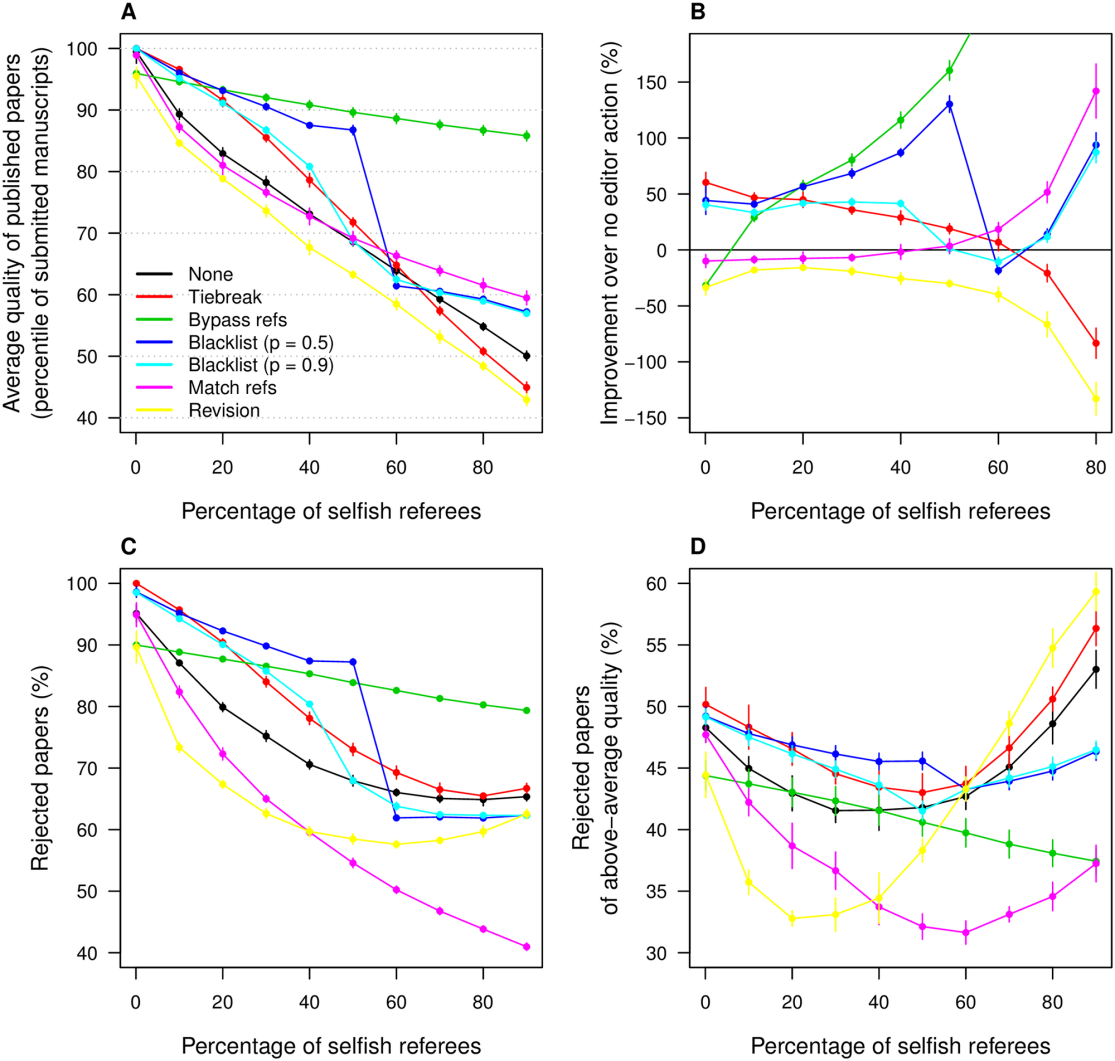
Effect of editor strategies. **A**: Average quality of accepted papers by percentage of selfish referees in the pool, measured in terms of the percentile of the quality distribution across submitted manuscripts. *None*: no editorial action; *Tiebreak*: if the paper is accepted by one referee but rejected by the other, the editor consults a third referee; *Bypass refs*: papers of exceptionally poor/good quality are automatically rejected/accepted by the editor without review; *Blacklist*: editor blacklists referees with a higher probability of being selfish than a threshold *p*. Results are shown for *p* = 0.5, 0.9. *Match refs*: editor pairs submitted manuscripts with referees of higher quality to avoid biased rejection from selfish referees; *Revision*: submitted papers with at least one rejection are sent back for revision and given a second round of reviews; **B**: Percentage improvement on average quality of published papers relative to no action. **C**: Percentage of rejected manuscripts by percentage of selfish referees in the pool. **D**: Percentage of rejected manuscripts with above-average quality. Color scheme in **B**, **C** and **D** identical to **A**.

None of the editorial strategies listed above could fully neutralize the decline in quality of published research caused by indifferent selfish referees (Fig 2A). However, some of these strategies were able to mitigate the impact.

Submitting contentious manuscripts to tiebreaking reviewers can raise the quality of accepted papers by as much as 10 percentile points of the distribution of submitted manuscripts (Fig 2A). Relative to no editorial action, it can represent an improvement of up to 50%, and the effect is positive as long as selfish referees do not comprise much more than half of the referee pool (Fig 2B). This makes sense, as a tiebreaking reviewer is more likely to be impartial than selfish in those circumstances. As selfish referees become so numerous as to predominate, the tiebreaking strategy becomes counterproductive. Tiebreaking also leads to more manuscript rejections overall (Fig 2C), and more rejection of above-average manuscripts in particular (Fig 2D).

We note that this strategy is equivalent to trying three referees from the beginning and deciding in favor of the majority. In S2 Fig we show results when three rather than two referees are consulted, in concert with our other editorial strategies. As with the tiebreaking scenario, three referees improve quality relative to two referees, as long as there are not too many selfish referees in the pool.

Bypassing reviewers in the case of exceptional papers (above the 90th quantile of submissions) and below-median papers has a strong impact, particularly for higher proportions of selfish referees (Fig 2A and 2C) where it strongly attenuates the decline in quality. Further testing reveals that most of this positive impact is due to automatic rejection of below-median papers. This is expected, as most papers are not of exceptional quality while many are of inferior quality. We note that when the presence of selfish referees is very small, this strategy backfires. This is because our moving-standard impartial referees have higher quality standards than our editors. While editors will accept anything above the 90th percentile, moving-standard referees will reject almost all but the very best submissions. The editor overrules some of these rejections, thus bringing quality down.

**Fig 3:**
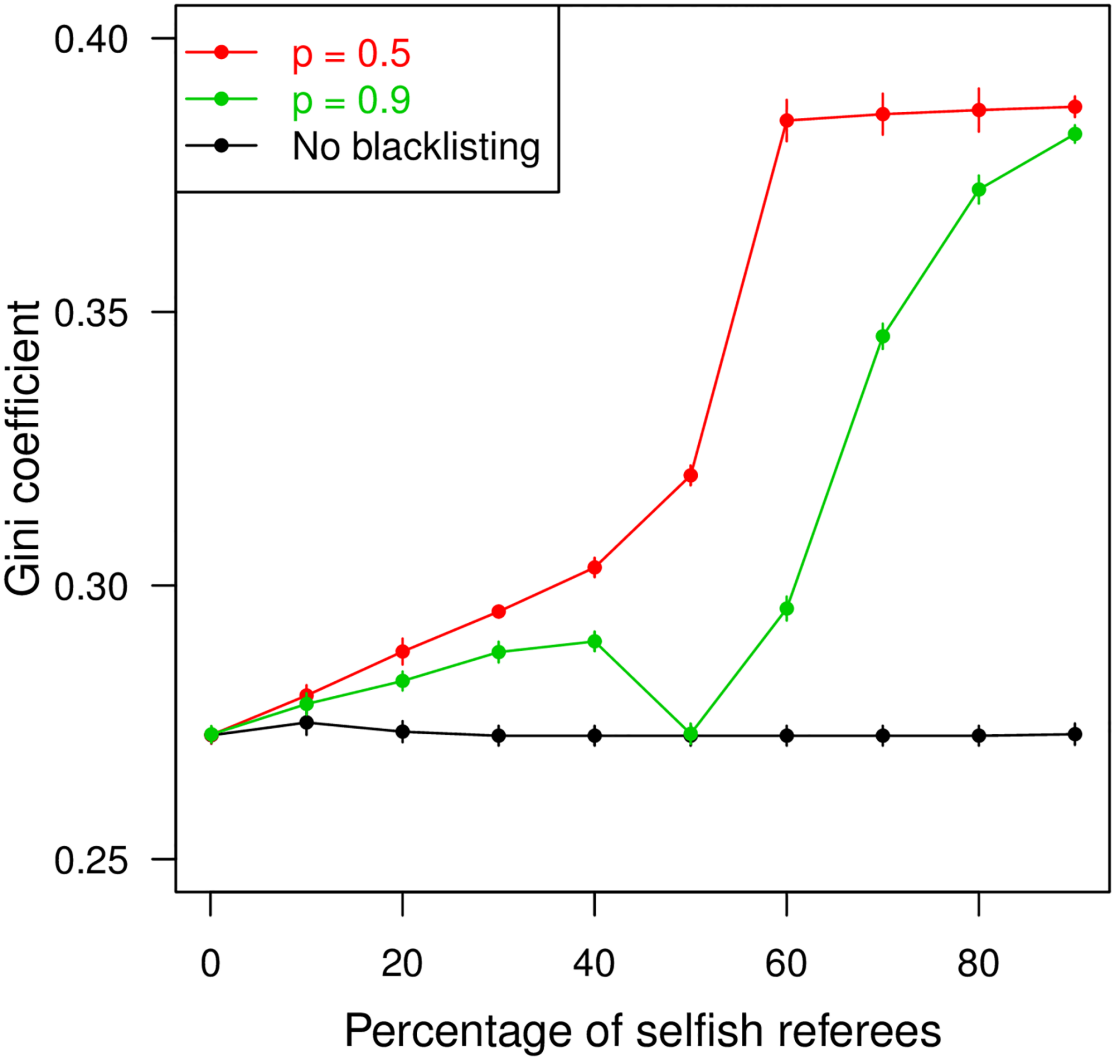
Evenness in referee load. Gini coefficient of the distribution of referee load plotted against percentage of selfish referees in the pool. Red and green curves show scenarios when editors blacklist referees with a higher than 50% and 90% chance of being selfish, respectively. Black curve shows Gini coefficient when editors do not blacklist. Referee load is defined as the number of times a scientist served as a referee. The Gini coefficient is half the average absolute difference in referee load across scientists, scaled by the average referee load of all scientists.

Blacklisting reviewers with a suspiciously high record of disagreements with other reviewers also improves quality of publications. The lower the probability threshold for blacklisting, the bigger the improvement (Fig 2A and 2B). Its effects on rejection statistics are similar to those of tiebreaking (Fig 2B and 2C). However, those benefits are paid for by higher inequality in the referee load across scientists: as some are excluded from the referee list, others must pick up the slack and serve as referees more frequently than they otherwise would. This is reflected as an increase in the Gini coefficient of the distribution of referee load (Fig 3). The impact is higher for a higher proportion of selfish referees in the pool, and for a lower blacklisting threshold.

Other editor strategies did not raise the quality of published papers relative to no action, although they reduced unwarranted rejections. Matching manuscripts to editors with a superior research output minimizes rejection based on a sense of threat or competition. This leads to much lower rejection rates, both in general (Fig 2C) and of above-average papers (Fig 2D). However, it does little to improve the quality of those papers that do get accepted, unless selfish referees vastly outnumber impartial ones (Fig 2A and 2B). This is because this strategy does not address the aspect of referee behavior that most impacts quality of published work, namely accepting low-quality papers.

Sending rejected manuscripts back for revision by the authors, a very common practice in many journals, does not help raise published quality either. In fact it lowers it, whether selfish referees are common or rare (Fig 2A and 2B). We explain this counterintuitive result thus: two indifferent selfish referees will only disagree if the manuscript is too good for one of them. Improving the quality of the manuscript will therefore not change the verdict of that referee, and might even make it too good for the other referee as well. Revising in this case does not help. A impartial and an indifferent selfish referee will disagree if the manuscript is either not good enough for the impartial referee or too good for the selfish referee. In the latter case revising does not change either review, and in the former it may work for the author, or it may overshoot the acceptance window of the selfish referee. Of course the original manuscript can be so bad that both referees reject it. In that case, revising may turn one or both reviewers around and get the paper in. Overall, revising is likely to make the most difference for papers of marginally acceptable quality, and their publication does little to boost the average quality of published work. More importantly, it does nothing to stop the acceptance of low quality work. What it does accomplish is a substantial decline in rejection of above-average manuscripts, particularly when selfish referees do not entirely dominate the pool (Fig 2D).

## Discussion

Cheating referees have been shown to severely lower the average quality of published literature [26]. We have shown that the brunt of this impact is due to their willingness to accept low-quality manuscripts, as opposed to their tendency to reject high-quality manuscripts perceived as competition. Published quality will remain high as long as referees commit to rejecting papers that fail to meet minimum standards set by journals, regardless of their potential inclination to sabotage work better than their own. Self-interested sabotaging has a relatively stronger impact on rejection rates, both in general and of above-average manuscripts.

We found that editors can mitigate, but not neutralize, the impact of indifference to quality by forwarding disputed manuscripts to tiebreaking referees, or alternatively by working with three referees rather than two. Our results corroborate previous findings that using a higher number of reviewers helps with quality insurance. However, a blanket strategy of using multiple reviewers has systemic costs in terms of time diverted from research activities towards reviewing [24]. By comparison, the tiebreaking strategy keeps such costs to a minimum. On the other hand, we note that effective strategies for maximizing quality also tend to increase the proportion of rejected manuscripts with above-average quality, especially if reviewers continually raise their standards.

Automatic acceptance or rejection without review of exceptional or inferior manuscripts had a strong positive impact on published quality, especially when the incidence of selfish referees is high. It also contributed to less rejection of above-average papers. Although this was one of the most potent strategies, it relies on the good judgment of editors regarding the quality of the manuscripts. In reality, specialized referees are often more qualified to judge technical soundness and novelty, while editors may have a better sense of the appeal of the manuscript to the journal’s readership. Of course, this strategy is vulnerable to the editors’ own biases, which were ignored in this study as we aimed to evaluate the maximum potential for good editors to counter bad referees.

Removing reviewers with a suspicious record from the referee pool increases quality. Grimaldo and Paolucci [28] found that blacklisting improves the quality of published research, minimizes disagreement between referees, and reduces rejection of good papers. However, they assumed that selfish referees share their concern for quality with impartial referees, and they only considered rejected papers that would have been accepted in the absence of selfish referees. Our results are consistent with [28], but we also found that blacklisting is less successful if selfish reviewers accept low-quality manuscripts, and we showed that under a broader definition of good papers that includes all manuscripts of above-average quality, blacklisting actually increases rejection of good papers. Furthermore, we emphasized that this editorial strategy increases the load on whitelisted referees to compensate for blacklisted referees. Because overburdened reviewers have less time for their own research [22], this ultimately punishes good referees.

Pairing manuscripts with good-quality reviewers and allowing authors to revise rejected manuscripts failed to improve quality relative to no action, but importantly both strategies lowered the proportion of above-average rejected manuscripts.

In sum, strategies that succeeded in improving quality did so at the expense of punishing authors of above-average papers or overtaxing good referees, while strategies that minimized these problems did little to improve quality. This conundrum highlights the need for policies going beyond chasing cheaters and strict screening for the best of the best. This is unlikely to be achieved without structural changes to align the interests of reviewers with those of peer review.

Our study made several simplifying assumptions. We assumed no referee bias other than a selfish interest in sabotaging competition. In reality, reviewers’ judgment may be colored by their professional or personal opinion of the authors, friendships, enmities, and other network effects. (This has led some journals to adopt a double-blind reviewing process, e.g. Nature, The American Naturalist, Social Science & Medicine). We also assumed editors have no bias of their own, although editor bias can have an even bigger impact on peer review than referee bias [25]. Differences in quality standards across different journals also affect the outcome of peer review models [28], but were left out of our study so we could focus on the impact of editorial strategies. Finally, we assumed that manuscripts are judged based on a single agreed-upon quantity that captures their scientific merit and publication worth. In reality, manuscripts are evaluated on several independent measures (novelty, clarity, accuracy, relevance). It would be interesting to see if our conclusions regarding the impact of referee indifference and the mitigating potential of editor strategies are borne out in more sophisticated models accounting for these omissions.

The success of blacklisting in our study bears emphasizing its limitations. As we pointed out, impartial referees never disagree and journals fully share information in our model. In reality, journals are more secretive, and honest referees disagree sometimes. Real-world editors must juggle between being flexible enough in their disagreement allowance to avoid blacklisting honest referees and strict enough to make sure cheating referees are removed from the pool. The strategy will still work as long as cheating referees disagree more often than impartial referees, albeit less effectively than under no honest disagreements. As for data-sharing across journals, full sharing maximizes the power of blacklisting. Partial or no sharing will correspondingly reduce it, as referees blacklisted from one journal can still be asked to review manuscripts for other journals. Systematic sharing of peer-review information could be more easily implemented by a group of journals managed by the same publisher and under the same submission/reviewing platform, as in the case of cascading reviews. Otherwise, initiatives such as the PEERE data-sharing protocol [3] may eventually help editors make informed decisions in referee selection. For now, however, there is no public repository of peer review data editors can count on.

On the other hand, some of our simplifications likely underestimate the efficiency of blacklisting. Our editors judge referees based solely on the number of disagreements. In reality, reviews consist of more than just accept/reject verdicts, and good editors may evaluate referees based on apparent conflicts of interest, timeliness, overall attitude, and the relevance, tone, thoroughness, and specificity of their reviews. Editors who judge referees holistically are likely in better position to find cheaters than editors who just count disagreements. Furthermore, we did not consider any reputation or professional costs to being blacklisted, such as increased difficulty in getting one’s own manuscript published. Recent proposals to match manuscripts to referees with similar reputation as the authors [32, 33] may discourage bad refereeing, thus enhancing the positive impact of blacklisting. Ultimately, the true potential of blacklisting will be better assessed with more refined models and empirical tests.

Finally, we note that several modifications to the classical peer review process have been proposed. Recent tests indicate that alternatives such as bidding and review-sharing may outperform classical peer review in speed of publication and quality control [30, 34]. It remains to be seen whether such alternatives are more robust to bias and cheating referee behavior.

In conclusion, peer review in its current format offers little incentive for altruistic behavior from referees, and has limited tools to safeguard the efficiency of the process. Efforts to minimize the number of bad papers accepted must balance the simultaneous goals of also minimizing the number of good papers rejected, and evenly distributing the burden of referee service among all scientists. While keeping in mind that ours is a very simple model, we suggest that editorial strategies can help, but a structural change that rewards good reviewing practices and discourages cheating may have a stronger impact. Although some say peer review is broken and in need of replacement [7, 8], most scholars still hold it in high regard and, while acknowledging the system’s flaws, advocate evidence-based efforts to improve it [18, 35]. In this latter context, future studies using more sophisticated models informed by empirical data should provide a better sense of the quantitative impact of referee and editor bias, and the most effective strategies to counter them.

## Supporting information

S1 Fig Effect of narcissistic referees. Narcissists accept only manuscripts that are similar enough to their own work to fall within the quality interval covering 95% of their own scientific production. These are meant to represent referees with a (conscious or unconscious) bias towards endorsing the relevance/importance of manuscripts on their subfield of expertise. Here we plot the effect of narcissistic referees on the quality of accepted (**A**) and rejected (**B**, **C**) papers, as a function of their percentage in the referee pool (the remainder being moving-standard impartial referees). For comparison, we also plot the effect of indifferent selfish referees (described in the main text).
(TIFF)

S2 Fig Two versus three referees. Average quality of accepted papers when two (**A**) and three (**B**) referees are assigned per manuscript, in concert with each editorial strategy tested in this study. Outcomes are qualitatively similar but quantitatively different. Three referees leads to better results overall (**C**), although not by very large percentage points, and the advantage declines with higher incidence of selfish referees in the pool, even reversing in some cases. *Q*_2(3)_ is the average quality of accepted papers under 2 (3) referees. Under three referees the editor always honors the majority vote, unless dictated otherwise by the editorial strategy at hand.
(TIFF)

S3 Fig Normal versus lognormal quality distribution. Average quality of accepted and rejected papers under normal (**A**, **B**, **C**) and lognormal (**D**, **E**, **F**) distribution of proficiency across authors and quality across a given author’s works. No editorial action considered. A normal distribution follows if manuscript quality is the end result of multiple random additive factors. A lognormal distribution occurs under multiplicative random factors. Comparison between the top and bottom rows indicates that our results are robust to relaxing the assumption of normality. Parameters: mean author proficiency 100 (normal, lognormal); standard deviation of proficiency 10 (normal), 0.5 (lognormal); standard deviation of quality per author’s works 5 (normal), 0.5 (lognormal).
(TIFF)

S1 Appendix Blacklisting referees. We provide the mathematical details of the editorial strategy of blacklisting referees with a high record of disagreements.
(PDF)
