# Supplementary figures and images for: Can editors save peer review from peer reviewers?
Source: PLoS One. 2017 Oct 9;12(10):e0186111. doi: 10.1371/journal.pone.0186111 (PMC5633172; doi:10.1371/journal.pone.0186111)

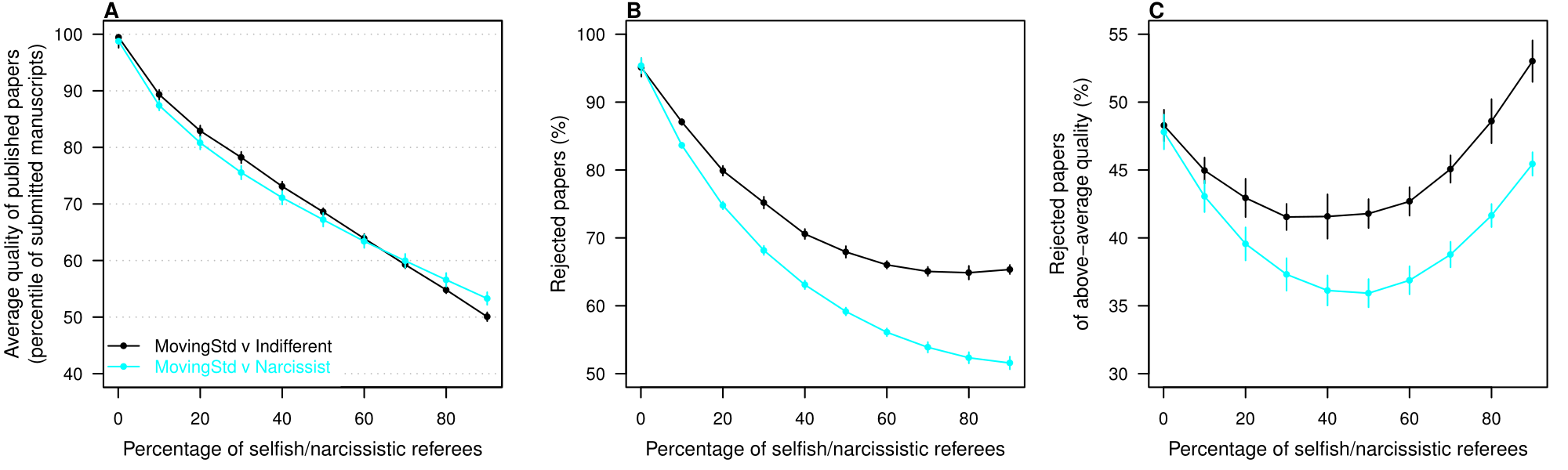

Supplement: S1 Fig — Narcissists accept only manuscripts that are similar enough to their own work to fall within the quality interval covering 95% of their own scientific production. These are meant to represent referees with a (conscious or unconscious) bias towards endorsing the relevance/importance of manuscripts on their subfield of expertise. Here we plot the effect of narcissistic referees on the quality of accepted (A) and rejected (B, C) papers, as a function of their percentage in the referee pool (the remainder being moving-standard impartial referees). For comparison, we also plot the effect of indifferent selfish referees (described in the main text). (TIFF) [file pone.0186111.s001.tiff]

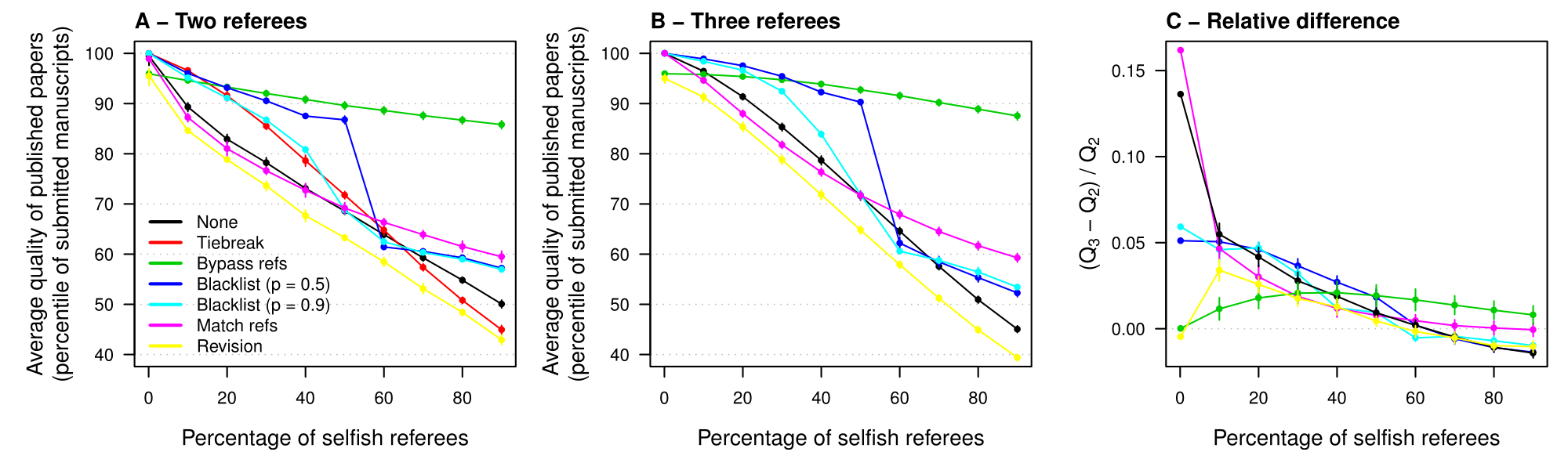

Supplement: S2 Fig — Average quality of accepted papers when two (A) and three (B) referees are assigned per manuscript, in concert with each editorial strategy tested in this study. Outcomes are qualitatively similar but quantitatively different. Three referees leads to better results overall (C), although not by very large percentage points, and the advantage declines with higher incidence of selfish referees in the pool, even reversing in some cases. Q2(3) is the average quality of accepted papers under 2 (3) referees. Under three referees the editor always honors the majority vote, unless dictated otherwise by the editorial strategy at hand. (TIFF) [file pone.0186111.s002.tiff]

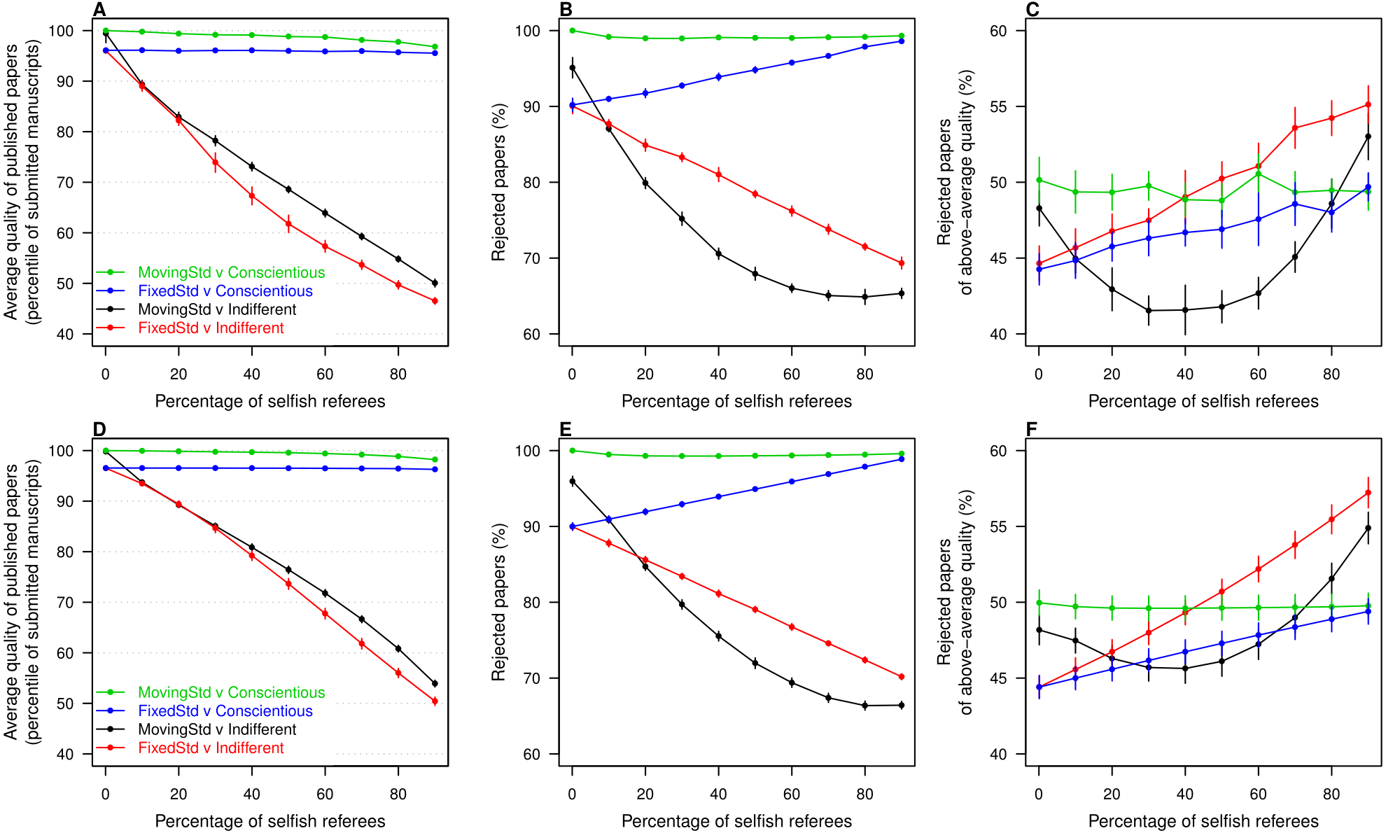

Supplement: S3 Fig — Average quality of accepted and rejected papers under normal (A, B, C) and lognormal (D, E, F) distribution of proficiency across authors and quality across a given author’s works. No editorial action considered. A normal distribution follows if manuscript quality is the end result of multiple random additive factors. A lognormal distribution occurs under multiplicative random factors. Comparison between the top and bottom rows indicates that our results are robust to relaxing the assumption of normality. Parameters: mean author proficiency 100 (normal, lognormal); standard deviation of proficiency 10 (normal), 0.5 (lognormal); standard deviation of quality per author’s works 5 (normal), 0.5 (lognormal). (TIFF) [file pone.0186111.s003.tiff]
