# Supplementary material for: Can editors save peer review from peer reviewers?
Source: PLoS One. 2017 Oct 9;12(10):e0186111. doi: 10.1371/journal.pone.0186111 (PMC5633172; doi:10.1371/journal.pone.0186111)

Can editors save peer review from peer reviewers?

Rafael D'Andrea[1,*] & James O'Dwyer[1]
[1]Dept. Plant Biology, University of Illinois at Urbana-Champaign, IL, USA
*rdandrea@illinois.edu

Appendix S1: Blacklisting referees with a suspicious record

Editors will blacklist any referee whose reviewing record indicates that they are likely to be selfish. impartial referees never disagree on their reviews, so a disagreement between two reviewers implies that at least one of them is selfish. A referee with a high record of disagreements is therefore likely to be selfish. Suppose editors blacklist referees whose probability of being selfish is equal or greater than p_0. We can calculate p_0 in terms of a referee's record of reviews and disagreements:

$$p_0 = P(S|k;n) = \frac{P(k|S;n)P(S)}{P(k|S;n) + P(k|D;n)} = \frac{f_s}{1 + \frac{P(k|D;n)}{P(k|S;n)}} \tag{1}$$

where $P(S|k;n)$ is the probability that a referee who has reviewed n papers and has a record of k disagreements is selfish, $P(k|S;n)$ is the probability that a selfish referee who has reviewed n papers will have a record of k disagreements (similarly for a impartial referee and $P(k|D;n)$), and $P(S)$ is the prior probability that the referee is selfish, which is simply the frequency of selfish referees in the pool, f_s.

The number of disagreements given the number of reviews is binomially distributed:

$$P(k|S;n) = \binom{n}{k} q_s^k (1-q_s)^{n-k} \tag{2}$$

$$P(k|D;n) = \binom{n}{k} q_d^k (1-q_d)^{n-k} \tag{3}$$

where q_s (q_d) is the probability that a selfish (impartial) referee will disagree with the other referee in a given review. Those in turn depend on the probability of disagreement between two selfish referees, q_{ss}, and the probability of disagreement between a selfish and a impartial referee, q_{sd} (two impartial referees never disagree).

$$q_s = f_s\, q_{ss} + f_d\, q_{sd} \tag{4}$$

$$q_d = f_s\, q_{sd} \tag{5}$$

where the terms are weighted by the probability that the other referee is selfish or impartial.

Two selfish referees will only disagree on a paper if the quality of that paper falls between the upper limits of their acceptance windows:

$$q_{ss} = \int_{Q_{\min}}^{\infty} \left(P_A(b_1) \int_{b_1}^{\infty} \left(P_A(b_2) \int_{b_1}^{b_2} P_P(x)dx \right) \mathrm{d}b_2 \right) \mathrm{d}b_1 \tag{6}$$

where $Q_{\min}$ is the lower limit of the acceptance window of all selfish referees, $P_P(x)\mathrm{d}x$ is the probability that the quality of a given submitted paper will fall between x and $x + \mathrm{d}x$, and $P_A(b)\mathrm{d}b$ is the probability that the quality of a given referee as an author will be between b and $b + \mathrm{d}b$. Equation 6 expresses the probability that the quality of the submitted paper falls between the upper limits of the acceptance windows of each selfish referee, integrated over all possible combinations of these upper limits (referee 2 is assumed, without loss of generality, to have the higher upper limit).

In contrast, a selfish referee and a impartial referee will agree if the paper falls either below the lower limit of acceptance of the selfish referee (in which case both will reject) or above the minimum imposed by the impartial referee but still below the upper limit imposed by the selfish referee (in which case both will accept). Otherwise they will disagree:

$$q_{sd} = 1 - \left(\int_{-\infty}^{Q_{\min}} P_P(x)\mathrm{d}x + \int_{\overline{Q}_{\mathrm{a}}}^{\infty} \left(P_A(b) \int_{\overline{Q}_{\mathrm{a}}}^{b} P_P(x)\mathrm{d}x \right) \mathrm{d}b \right) \tag{7}$$

where $\overline{Q}_{\mathrm{a}}$, the average quality of accepted papers in stationary equilibrium, is the minimum quality accepted by impartial referees. This quantity is a function of the proportion of selfish referees in the pool, and we calculate it numerically[1]. As described in the main text, the distribution of authors is $N(100, \sigma^2_{\mathrm{author}})$, and the distribution of submitted papers is $N(100, \sigma^2_{\mathrm{author}} + \sigma^2_{\mathrm{quality}})$:

$$P_P(x) = \frac{1}{\sqrt{2(\sigma^2_{\mathrm{author}} + \sigma^2_{\mathrm{quality}})\pi}} \exp\left(-\frac{(x-100)^2}{2(\sigma^2_{\mathrm{author}} + \sigma^2_{\mathrm{quality}})} \right) \tag{8}$$

$$P_A(b) = \frac{1}{\sqrt{2\sigma^2_{\mathrm{author}}\pi}} \exp\left(-\frac{(b-100)^2}{2\sigma^2_{\mathrm{author}}} \right) \tag{9}$$

Figure 1 shows the minimum number of disagreements in a referee's record required for blacklisting as a function of the number of papers the referee has reviewed, obtained by numerically evaluating Equation 1. Notice that the number of disagreements needed for blacklisting is roughly proportional to the number of reviews, and is typically close to half of the number of reviews whether selfish referees are as rare as 10% or as common as 40% of the referee pool. We use a cutoff $p_0 = 0.9$, but results are insensitive to that choice; in fact, p_0 can be as low as 0.5 without changes to the figure. This is because $P(S|k;n)$ rises abruptly with k, from close to 0 below the threshold to close to 1 above it.

[1] Strictly speaking, $\overline{Q}_{\mathrm{a}}(f_s, t)$ varies in time and is affected by blacklisting. However, q_{sd} is reasonably insensitive to the exact value of $\overline{Q}_{\mathrm{a}}$, so for simplicity we use the stationary value $\overline{Q}_{\mathrm{a}}(f_s, \infty)$ observed under no blacklisting.

Figure 1: Minimum number of disagreements on a referee's reviewing record given the number of papers reviewed that indicate higher than 90% probability that the referee is selfish. Referees remain in the pool for 35 years, or 70 reviewing cycles, and then are replaced by new referees.

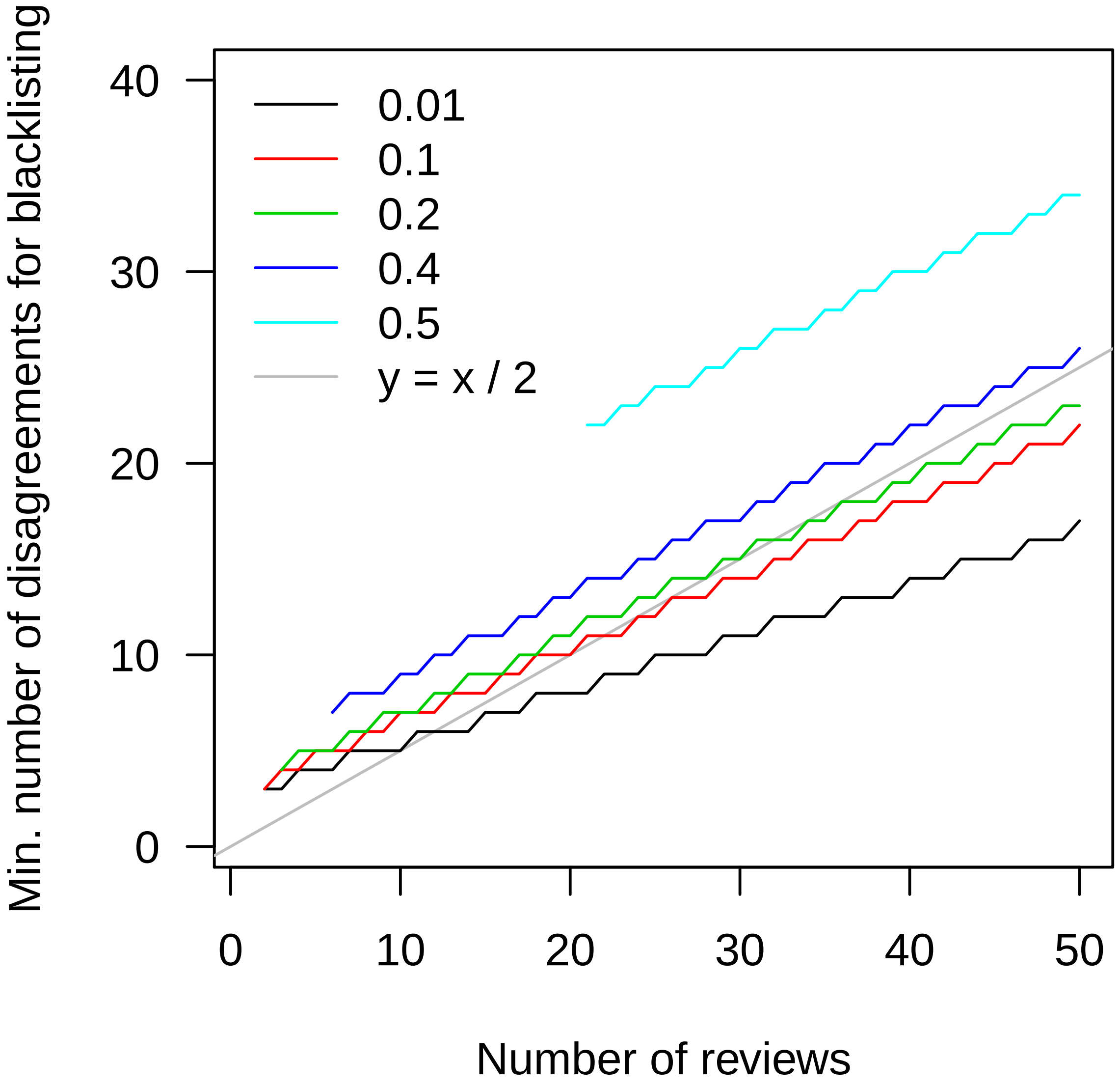

Supplement: S1 Appendix — We provide the mathematical details of the editorial strategy of blacklisting referees with a high record of disagreements. (PDF) [file pone.0186111.s004.pdf]
